# Head and neck squamous cell carcinoma in adults aged 45 or younger—an analysis of two tertiary cancer centres

**DOI:** 10.1007/s00066-025-02448-2

**Published:** 2025-08-21

**Authors:** Charlotte Frei, Soeren Schnellhardt, Sabine Semrau, Sarina K. Mueller, Manuel Weber, Justus Kaufmann, Rainer Fietkau, Sophia Drabke, Marlen Haderlein

**Affiliations:** 1 Department of Radiation Oncology, Universitätsklinikum Erlangen, Friedrich-Alexander-Universität Erlangen-Nürnberg, Erlangen, Germany; 2 Department of Radiotherapy and Radiation Oncology, Saarland University Medical Center, Homburg, Germany; 3 Department of Otorhinolaryngology—Head and Neck Surgery, Universitätsklinikum Erlangen, Friedrich-Alexander-Universität Erlangen-Nürnberg, Erlangen, Germany; 4 Department of Oral and Maxillofacial Surgery, Universitätsklinikum Erlangen, Friedrich-Alexander-Universität Erlangen-Nürnberg, Erlangen, Germany; 5 Department of Radiation Oncology, Universitätsklinikum Mainz, Mainz, Germany; 6 Comprehensive Cancer Center Erlangen-EMN, Erlangen, Germany

**Keywords:** HNSCC, Young patients, Radio(chemo)therapy, Recurrence patterns, Risk factors

## Abstract

**Purpose:**

The aim of this study was to evaluate the outcome, especially disease-free survival (DFS) and recurrence patterns, in patients with a maximum age of 45 years at first diagnosis of head and neck squamous cell carcinoma (HNSCC).

**Methods:**

We retrospectively reviewed data from 79 patients with newly diagnosed HNSCC aged 45 or younger without distant metastasis who underwent postoperative or definitive radio(chemo)therapy in either the Department of Radiation Oncology at the University Hospital of Erlangen or the Department of Radiation Oncology at the University Hospital of Mainz between September 2006 and December 2023. The Kaplan–Meier method was used to calculate survival and recurrence rates. In univariate analysis, the log-rank test was used to correlate patient-/tumour- and treatment-related parameters to survival and recurrence rates.

**Results:**

The overall survival rate was 79.7% at 2 years and 67.1% at 5 years. The DFS rate was 73.4% at 2 years and 67.1% at 5 years. Cumulatively, 14.6% of patients in the postoperative arm had locoregional recurrences at 2 years and 23.0% at 5 years, while 25.7% of patients in the definitive arm had local recurrences at 2 years and 33.1% at 5 years (*p* = 0.36). The rate of distant metastasis was 19.2% in the postoperative arm at 2 years and 21.6% at 5 years. In the definitive arm, the distant metastasis rate was 20.7% at 2 years and 28.6% at 5 years (*p* = 0.49). Disease-free survival was significantly improved in patients who drank little or no alcohol (*p* = 0.005) and in patients with a low UICC stage (*p* < 0.001). No differences in DFS were observed for different primary tumour locations or treatment modalities.

**Conclusion:**

Locoregional recurrences were the most common site of recurrence, regardless of tumour location and treatment modality. Therefore, future study designs in this patient cohort should potentially investigate intensified treatment approaches.

## Introduction

In 2020, 4560 women and 11,830 men were diagnosed with head and neck cancer in Germany [1]. Head and neck tumours encompass a diverse range of cancers that originate in various anatomical locations and have various histologies. The term “head and neck cancer” typically refers to cancers located in the oropharynx, hypopharynx, oral cavity and larynx. The most common histological subtype is squamous cell carcinoma. The incidence is stable in women and slightly decreasing in men. The 5‑year survival rate for women is 64% and for men 52% in oropharyngeal, oral cavity and hypopharyngeal cancers and 64% for both genders in laryngeal cancer.

The average age of onset is between 65 and 75 years. The primary treatment for patients diagnosed with head and neck squamous cell carcinoma (HNSCC) is either surgery followed by adjuvant radio(chemo)therapy or without any adjuvant treatment depending on T and N stages or primary radiochemotherapy.

HNSCC is often associated with consumption of tobacco and alcohol. Another risk factor is chronic infection with subtypes of the human papillomavirus (HPV) [1], whereby HPV-related oropharyngeal cancer is associated with better disease-free survival (DFS) [2].

We were interested in the outcomes and risk factors of adults aged younger than 45 years because on the one hand, long-term exposure to tobacco and alcohol in this group is rare, and on the other, DFS rates seemed not to be better than in older patients [3]. The incidence in this group compared to the overall incidence of HNSCC is low, with 4%–6% of patients with oral cancer aged younger than 40 years [4]. Oral cancer accounts for 6.7% of all cancers in patients under the age of 45 [5].

Understanding the prognostic factors that influence survival in this age group is crucial, as it can guide clinical decision-making and treatment strategies. Prior research has shown that younger patients may have different tumour biology, treatment responses, and psychosocial needs, which necessitates a tailored approach to their care [6].

The aim of this retrospective analysis was to investigate survival data, recurrence patterns, treatments strategies and patient-related risk factors.

## Materials and methods

In this retrospective analysis, we examined a cohort of 79 patients diagnosed with HNSCC who were treated with radio(chemo)therapy in two tertiary cancer centres in Germany, either in the Department of Radiation Oncology of the University Hospital of Erlangen or the Department of Radiation Oncology of the University Hospital of Mainz, between September 2006 and December 2023. By utilizing the Kaplan–Meier method for survival analysis, we aimed to identify correlations between patient and tumour characteristics, treatment modalities, tumour sites, and outcomes. The findings of this study will contribute to the understanding of HNSCC in younger patients and may inform future clinical practices to improve their prognosis.

### Patient characteristics

We reviewed the clinical records of patients aged younger than 45 years with newly diagnosed HNSCC. Exclusion criterion was the presence of distant metastases. For detailed patient information, see Table 1.

**Table 1:** Patient characteristics

Patient characteristics (*N* = 79)		
Characteristic	Value	%
*Gender (number of patients)*		
Male	50	63.3
Female	29	36.7
*Age at diagnosis (years)*		
Median	41	–
Range	23–45	–
*Smoking (n)*		
Smoker/former smoker	56	70.8
Non-smoker	21	26.6
Not specified	2	2.5
*Alcohol (n)*		
Alcohol abuse/former alcohol abuse	25	31.7
Occasional consumption of alcohol	25	31.6
No consumption of alcohol	27	34.2
Not specified	2	2.5
*Primary tumour site (n)*		
Oropharynx	24	30.4
HPV positive	7	8.8
HPV negative	5	6.3
Not specified	12	15.3
Oral cavity	37	46.8
Larynx	7	8.9
Hypopharynx	9	11.4
Cervical cancer of unknown primary	2	2.6
*Therapeutic concept (n)*		
Primary radiochemotherapy	29	36.7
Postoperative radio(chemo)therapy	50	63.3
Tumour stage (n)*		
cT1	16	20.3
cT2	26	32.9
cT3	15	19.0
cT4	20	25.3
cT0/x	2	2.6
Nodal stage (n)*		
cN0	19	24.1
cN1	13	16.5
cN2	39	49.4
cN3	8	10.1
*UICC overall collective (n)*		
I	8	10.1
II	10	12.7
III	14	17.7
IVA	39	49.4
IVB	8	10.1
*UICC (Union Internationale Contre le Cancer) postoperative collective (n)*		
I	5	10.0
II	8	16.0
III	10	20.0
IVA	24	48.0
IVB	3	6.0
*UICC definitive collective (n)*		
I	3	10.3
II	2	6.9
III	3	10.3
IVA	15	51.7
IVB	3	10.3
*Grading (n)*		
G1	4	5.1
G2	36	45.6
G3	30	38.0
Not specified, HPV+	3	3.7
Not specified	6	7.6

*TNM AJCC (American Joint Committee on Cancer) 7th edition

### Treatment

#### Radiotherapy

All patients received radio(chemo)therapy, either in a primary (36.7%) or an adjuvant (63.3%) setting. Two patients were treated with brachytherapy alone (2.5%), 71 patients with teletherapy alone (89.9%) and six patients were treated with teletherapy followed by a boost with brachytherapy (7.6%). Teletherapy involved the application of different techniques, including 3D-CRT (Three-Dimensional Conformal Radiation Therapy), IMRT (Intensity Modulated Radiation Therapy) and VMAT (Volumetric Modulated Arc Therapy).

The median radiotherapy dose applied in the adjuvant setting by teletherapy alone was 64 Gy (Gray) (range: 54 to 70 Gy), with one patient cancelling treatment after 10 Gy. Radiotherapy was administered daily, five times a week, with a single fraction dose of 2 Gy. The patient who received 70 Gy developed a relapse during radiotherapy treatment. One patient treated with brachytherapy alone in the adjuvant setting received HDR (High Dose Rate) brachytherapy with a single fraction dose of 3.8 Gy and a cumulative dose of 38 Gy. Three patients were treated with teletherapy to a cumulative dose of 50 or 56 Gy combined with a PDR (Pulsed Dose Rate) brachytherapy boost consisting of a single pulse dose of 0.5–0.6 Gy to a cumulative dose of 10 to 12 Gy.

The median radiotherapy dose in the definitive setting was 70 Gy in primary tumour regions and in involved lymph nodes (range: 60 to 73.4 Gy); 22 out of 29 patients (75.9%) received a dose of 70 Gy or higher. Three patients with laryngeal cancer UICC stage I received 64 or 66 Gy, and another three patients received 64 Gy as part of a clinical trial. The dose was either applied through daily radiotherapy, five times a week, with a single fraction dose of 2 Gy, or through bidaily radiotherapy, five days a week, with a single fraction dose of 1.4 Gy. In elective lymphatic drainage areas, 50 to 60 Gy was applied. One patient received PDR brachytherapy at the primary tumour site alone, with a single pulse dose of 0.57 Gy to a cumulative dose of 60 Gy. Three patients received teletherapy combined with a brachytherapy boost to the primary tumour site, with a cumulative dose of 70–73.4 Gy.

#### Chemotherapy

All patients undergoing definitive percutaneous radiotherapy received concomitant chemotherapy. In the postoperative group, 29 out of 48 patients (60.4%) received chemotherapy. The indication for postoperative chemotherapy was based on extracapsular spread (24.1%), close resection margins (13.8%), involvement of three or more lymph nodes (17.2%), or the individual decision of the treating physicians (65.5%). The following chemotherapy regimens were used in both definitive and adjuvant settings: 34 patients received platinum + 5-fluorouracil (61.8%), 11 received platinum monotherapy (20.0%) and 10 received platinum + paclitaxel (18.1%).

### Toxicity data

In the group of patients who survived for 2 years after treatment, we evaluated long-term toxicity. This group included a total of 54 patients. Five patients were lost to follow-up, and no data could be obtained for them. Long-term toxicity was assessed using the Common Terminology Criteria for Adverse Events (CTCAE), versions 3 to 5, depending on the year of evaluation. For detailed toxicity data, see Table 2.

**Table 2:** Toxicity data

Toxicity data (*N* = 49)		
Toxicity	Value	%
*Pre-radiotherapy nutrition*		
Normal nutrition	34	69.4
Pureed/soft food	9	18.4
Supplemental nutrition via PEG	5	10.2
Nutrition via PEG only	1	2.0
*Long-term nutrition*		
Normal nutrition	36	73.5
Pureed/soft food	11	22.4
Supplemental nutrition via PEG	0	0
Nutrition via PEG only	2	4.1
*Xerostomia*		
Grade 0	9	18.4
Grade 1	29	59.2
Grade 2	11	22.4
Grade 3	0	0
*Dysphagia*		
Grade 0	19	38.8
Grade 1	16	32.7
Grade 2	9	18.4
Grade 3	5	10.2
Grade 4	0	0
*Voice alteration*		
Grade 0	32	65.3
Grade 1	14	28.6
Grade 2	1	2.0
Grade 3	2	4.1
*Oesophageal stricture*		
Grade 0	30	61.2
Grade 1	14	28.6
Grade 2	4	8.2
Grade 3	1	2.0
Grade 4	0	0
*Trismus*		
Grade 0	39	79.6
Grade 1	5	10.2
Grade 2	5	10.2
Grade 3	0	0
*Osteoradionecrosis*		
No	44	89.9
Yes, and the initial tumour resection involved surgery on the jawbone	3	6.1
Yes, and the initial tumour resection did not involve surgery on the jawbone	2	4.1

### Data analysis and statistics

Overall survival, disease-free survival, and the cumulative incidence of locoregional recurrence and distant metastases were measured from the time of first diagnosis (defined as the date of biopsy). Disease-free survival was defined as the absence of locoregional or distant disease recurrence or death. Statistical analysis was performed using SPSS software version 28 (IBM Corp., Armonk, NY, USA). The Kaplan–Meier method was used to estimate overall survival, disease-free survival, and the cumulative incidence of local/locoregional control and distant metastases. In univariate analysis, the log-rank test was employed to correlate patient-, tumour-, and treatment-related parameters with overall survival, disease-free survival, and the incidence of local/locoregional recurrences and distant metastases.

## Results

The median follow-up was 52.8 months (minimum 1 month; maximum 158 months). The patient with the minimum follow-up of 1 month developed both locoregional and distant progression after postoperative radiochemotherapy in UICC stage IVB.

Patients receiving adjuvant radio(chemo)therapy began radiotherapy a median of 53.3 days (minimum 14 days; maximum 125 days) after tumour resection. Taking re-resected cases into account, the median time to start radiotherapy was 47.8 days (minimum 14 days; maximum 91 days) after the last resection.

### Overall survival

**Fig. 1:**
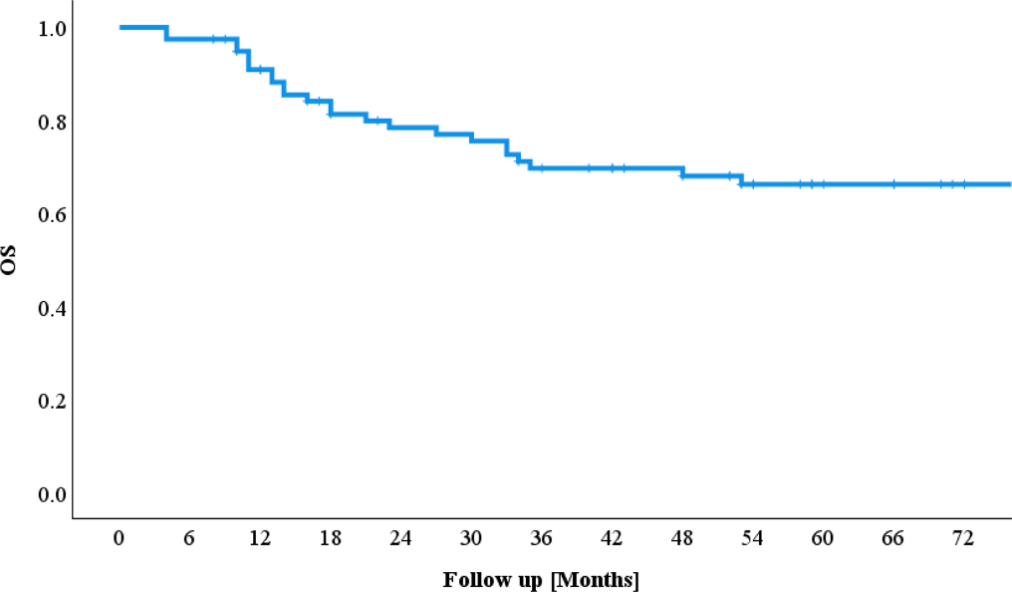
Overall survival (*OS*)

The overall survival (OS) rate was 79.7% after 2 years and 67.1% after 5 years; 65.8% of the patients were still alive at the end of the follow-up period. The minimum overall survival was 4 months, and the maximum follow-up duration ended after 160 months (Fig. 1).

Considering the UICC stage and divided into definitive and postoperative treatment cohorts, the OS rates were as follows: patients with UICC stage I and II disease showed survival rates of 75% after 2 and 5 years in the definitive treatment arm (*n* = 6), and survival rates of 100% after 2 and 5 years in the adjuvant treatment arm (*n* = 12). Patients with UICC stage III and IVA disease receiving definitive radiochemotherapy (*n* = 17) showed survival rates of 75.3% after 2 years and 50.3% after 5 years, while those receiving adjuvant radio(chemo)therapy (*n* = 36) showed survival rates of 86% after 2 years and 74.2% after 5 years. In UICC stage IVB, survival rates were 20% after 2 and 5 years in the definitive treatment group (*n* = 5) and 33.3% after 2 years and 0% after 5 years in the adjuvant group (*n* = 3).

### Disease-free survival

**Fig. 2:**
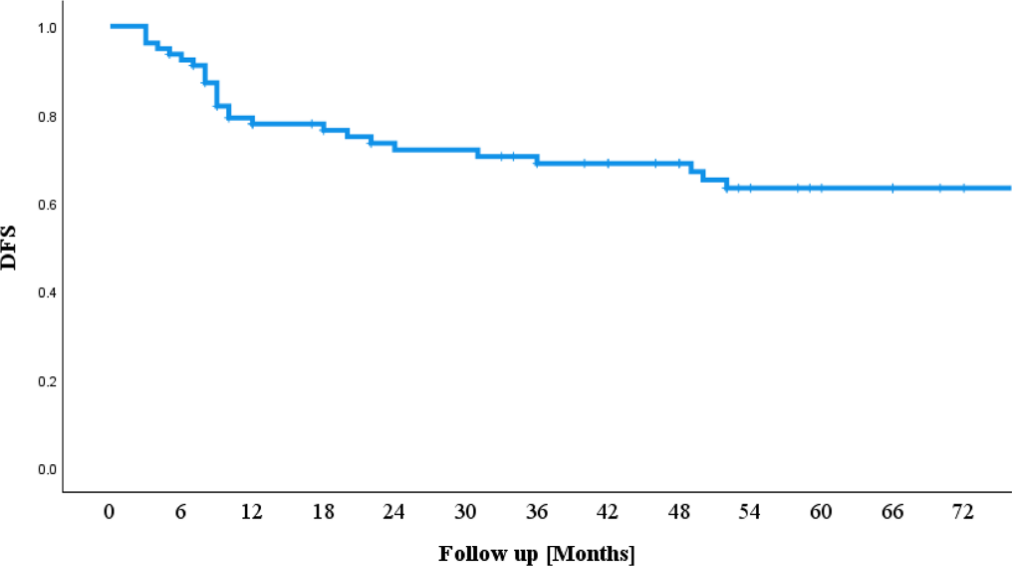
Disease-free survival (*DFS*)

The DFS rate was 73.4% after 2 years and 67.1% after 5 years; 65.8% of the patients did not develop a relapse by the end of follow-up (Fig. 2).

Patients receiving definitive radiochemotherapy showed a DFS of 64.2% after 2 years and 51.4% after 5 years. In the postoperative group, DFS was 76.6% after 2 years and 69.3% after 5 years. (*p* = 0.18). Among patients receiving adjuvant treatment within 42 days after tumour resection, DFS was 77.9% after 2 years and 68.2% after 5 years. For those receiving postoperative treatment outside this interval, DFS was 71.5% after 2 years and 65.2% after 5 years (*p* = 0.87).

**Fig. 3:**
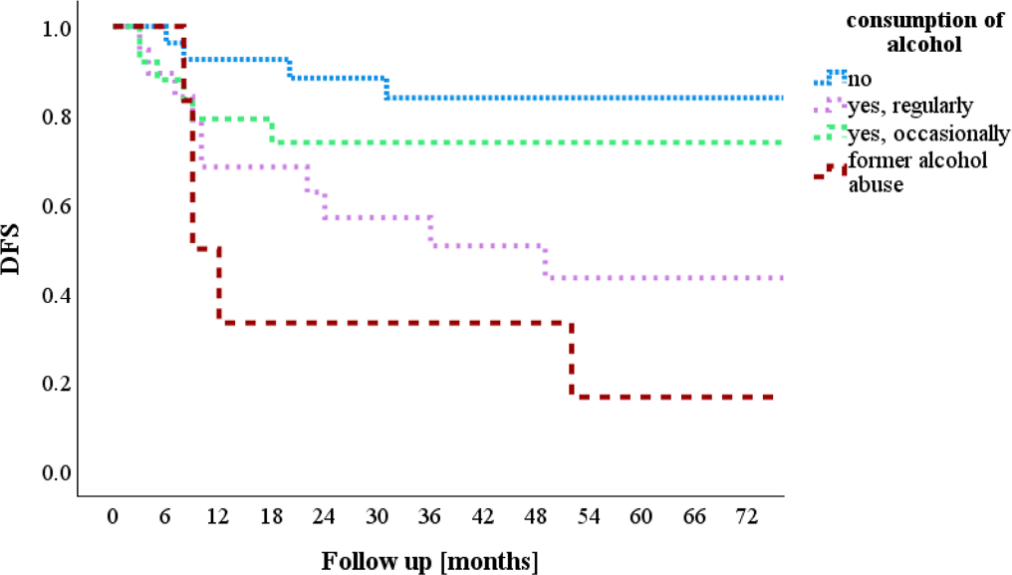
Alcohol consumption and disease-free survival (*DFS*)

Few parameters were found to correlate significantly with DFS. Firstly, alcohol consumption was significantly correlated with DFS (Fig. 3). Twenty-seven patients (34.2%) reported not consuming alcohol, and this group had a DFS of 84.0%. In contrast, six patients (7.6%) with a history of alcohol abuse showed a DFS of only 16.7% (*p* = 0.005).

**Fig. 4:**
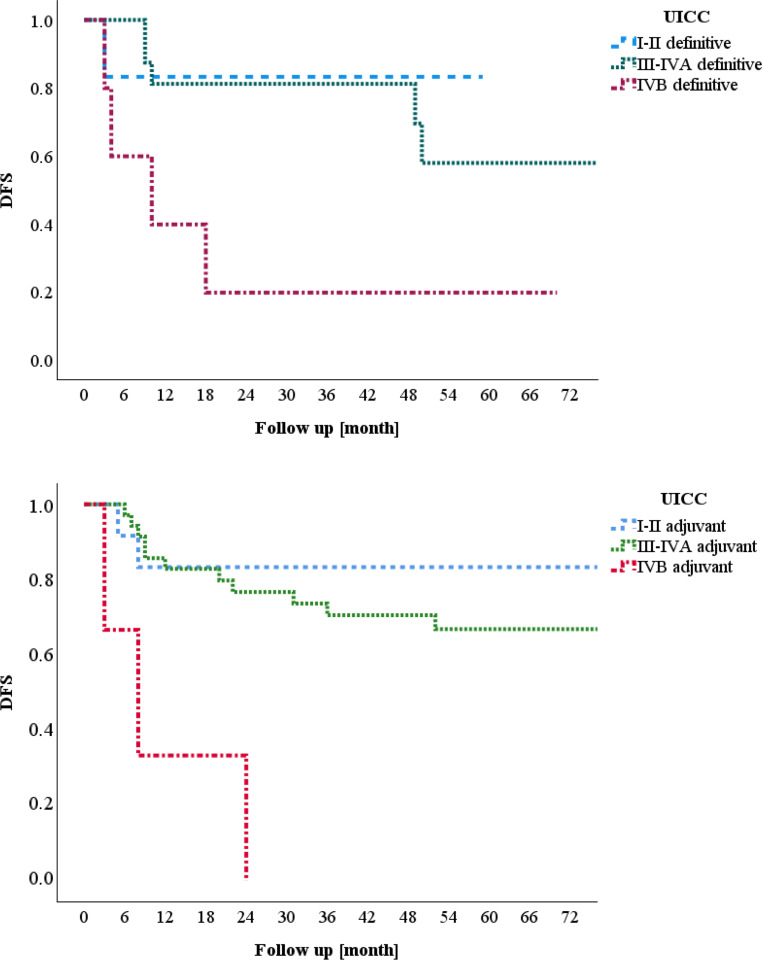
Union for International Cancer Control (*UICC*) stage and disease-free survival (*DFS*)

Considering the UICC stage, DFS rates were as follows (Fig. 4): in the group treated with definitive radiochemotherapy, DFS after 5 years was 83.3% for patients included in UICC I–II. Out of six patients, only one suffered a relapse, and this occurred only 3 months after treatment. Seventeen patients were classified into UICC III–IVA, with a DFS of 81.3% after 2 years and 58% after 5 years. Five patients were included in UICC IVB, and four of them suffered a relapse, resulting in a DFS rate of 20.0% after 2 years (*p* < 0.001).

In the cohort of patients treated with adjuvant radio(chemo)therapy, we observed slightly different values. In UICC I–II, DFS was also 83.3% after 5 years. Out of 12 patients, 2 suffered a relapse within the first year after treatment. One patient suffered a relapse 8 years after first diagnosis. In UICC III–IVA, 36 patients were included, with a DFS of 76.7% after 2 years and 66.9% after 5 years. In UICC IVB, 3 patients were included, resulting in a DFS of 0% after 2 years.

We did not find significant results regarding DFS when considering other risk factors such as smoking, positive family history, a long time between surgery and adjuvant radio(chemo)therapy, or histopathologic characteristics (e.g. perineural invasion, lymphovascular invasion, grading). We also did not find significant results regarding DFS when considering different tumour sites.

**Fig. 5:**
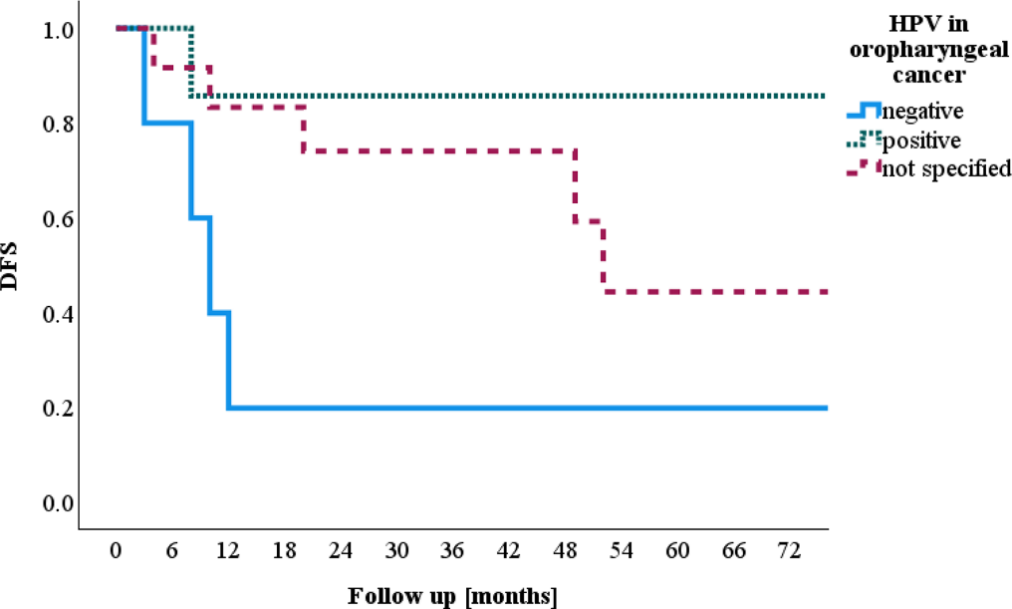
Human papillomavirus (*HPV*) in oropharyngeal cancer and disease-free survival (*DFS*)

In the subgroup of oropharyngeal cancer, there were 24 patients (Fig. 5). The HPV status was not determined in this cohort before 2015. HPV status was determined in 12 patients, of whom 7 were diagnosed with HPV-positive oropharyngeal cancer, resulting in a DFS of 85.7% after 5 years. Five patients were HPV negative, with a DFS of 20% after 5 years. In the group of oropharyngeal cancer patients without HPV status determination, the DFS was 44.4% after 5 years (*p* = 0.09).

### Locoregional recurrence

Cumulatively, 12.0% of patients in the postoperative arm had locoregional recurrences at 2 years and 23.0% at 5 years, while 25.7% of patients in the definitive arm had locoregional recurrences at 2 years and 33.1% at 5 years (*p* = 0.36). In patients receiving postoperative radiochemotherapy, the locoregional recurrence rate was 10.6% after 2 years and 21.5% after 5 years, compared to patients receiving postoperative radiotherapy alone, who had a recurrence rate of 14.6% after 2 years and 25.8% after 5 years (*p* = 0.5). After 5 years, 38.9% of locoregional recurrences occurred at the primary tumour site only, 27.8% in the lymphatic drainage area only, and 33.3% at both the primary tumour site and in the lymphatic area. No significant differences in recurrence patterns were observed between treatment modalities (*p* = 0.9). Both locoregional recurrence and distant metastasis was developed by 33.3% .

### Distant metastasis

The rate of distant metastasis was 19.2% in the postoperative arm at 2 years and 21.6% at 5 years. In the definitive arm, the distant metastasis rate was 20.7% at 2 years and 28.6% at 5 years (*p* = 0.49).

### Second cancer

Four patients developed a second malignancy (5.1%). Three of them developed oesophageal cancer, and one developed lung cancer. One patient developed a second malignancy 3 years after the first diagnosis, two patients developed it 6 years after diagnosis, and one patient developed it 12 years after diagnosis. The cumulative incidence after 5 years was 1.9%, and after 10 years it was 9.2%.

## Discussion

This study evaluated the outcomes of patients aged 45 years or younger with HNSCC. Because of the specific nature of this cohort, our study has several limitations. First, it is a retrospective analysis covering a period of 17 years. Despite this long period, only 79 patients could be included. Due to this extended timeframe, there is a lack of differentiation regarding HPV status, as HPV differentiation started only in 2015 in our cohort. Additionally, due to the retrospective nature of the study, toxicity data for some patients and follow-up information are partially inconsistent, and several treatment regimens and techniques were used. Furthermore, the small sample size in our analysis must be taken into account; as a result, some subgroups were so small that no statistically significant results could be expected.

To summarize, the overall and disease-free survival rates were similar to those of the overall patient population suffering from HNSCC. In the KEYNOTE-412 trial, which considered patients with locally advanced HNSCC in definitive treatment, DFS after 24 months was 56% in the control group, and OS was 72% after 36 months in the control group [7]. In the KEYNOTE-412 study, the median age in the control arm was 58.8 years, considering 402 patients. Disease-free survival was comparable to our cohort, which demonstrated a slightly higher DFS of 67.4% in the definitive treatment group with UICC stage III–IVB, hence considering 22 patients only. There is a disagreement in the literature regarding young patients with HNSCC, and most reviews consider cases from the past century [8–13]. For instance, Sarkaria et al. [10] reported worse outcomes for patients with tongue cancer aged under 40, with DFS rates < 43%. However, this was a retrospective analysis of 132 patients diagnosed between 1968 and 1993. In contrast, Friedlander et al. showed a DFS rate of 62% in patients with tongue cancer under 40 treated between 1984 and 1993 [11], and Mukdad et al. reported DFS rates > 70% in patients treated between 1973 and 2012 [9]. Thus, one might assume that differences in survival rates are influenced by advancements in treatment.

Despite the young age, alcohol consumption remains an important risk factor in our cohort. Other authors have suggested that patients under 40 years of age are unlikely to have a history of alcohol or tobacco abuse and that the duration of exposure would be too short for malignant transformation to occur [14]. Although we did not find a significant result for tobacco, one might assume that both alcohol and tobacco are the most common risk factors for HPV-negative HNSCC, regardless of age [15]. In our cohort, 70% of patients were smokers.

Other authors discuss an increased mutagen sensitivity in cancer development and the risk of developing a secondary malignancy in young patients [16]. In our cohort, the cumulative incidence of secondary malignancies after 10 years was 9.2%, which is similar to what is typically reported in the literature across all age groups [17–19]. For example, Lu et al. [19] reported a rate of 9.4% for metachronous second primary cancer in a meta-analysis of patients suffering from HNSCC. Therefore, we cannot assume an increased mutagen sensitivity in our cohort despite the young age, although there was not a uniform follow-up period.

Unsurprisingly, we observed better DFS in patients with HPV-positive oropharyngeal cancer. However, to achieve more significant results, a larger number of cases would be needed. Additionally, further molecular analysis of high- and low-risk HPV subtypes could lead to more personalized treatment recommendations [20].

The outcome for patients receiving adjuvant radio(chemo)therapy in UICC stages I and II was similar to that in patients receiving definitive radiochemotherapy, with a DFS of 83.3% after 5 years. It remains unclear—also due to the small sample size—whether a treatment delay, such as one caused by wound healing complications, in adjuvant treatment after surgery has an impact on outcome. This potential effect has been discussed by several authors with contrasting results; however, most emphasize the importance of an appropriate adjuvant treatment. For example, Huang et al. [21] reported the impact of delayed treatment in breast cancer and HNSCC in a systematic review of 46 studies, while Suwinsky et al. [22] suggested that treatment gaps during radiotherapy might be a more significant factor than gaps between surgery and radiotherapy. Additionally, Marshak et al. [23] and Balk et al. [24] found no significant impact of delayed adjuvant treatment. Based on these data, in conjunction with toxicity data, consideration should be given to whether patients would benefit from combination therapy or if the rapid initiation of definitive radiochemotherapy would be more advantageous.

In the locally and nodally advanced stages (UICC III and IVA), we could not identify an optimal treatment strategy, although the cumulative DFS rates using the Kaplan–Meier method were slightly better in the postoperative treatment arm, with a DFS of 66.9% after 5 years compared to 58.0%. However, this difference is not considered statistically significant. The literature also presents an ongoing debate regarding the optimal treatment for these stages [25]. The prognosis in UICC stage IVB is very poor, regardless of the chosen treatment. In our cohort, only 8 patients were included in this subgroup, with just one long-term survivor who was treated with definitive radiochemotherapy. The size of our cohort remains insufficient for a valid comparison of the two treatment strategies.

Regarding recurrence patterns, most recurrences occur locoregionally. Locoregional recurrence rates of about 25% contribute to the poor outcomes. Therefore, in the future, optimized multimodal treatment options need to be established.

## Conclusion

In this analysis, the prognosis of young patients with HNSCC is not worse than that of older patients in historical control groups. The specific risk factors for young patients remain unclear. Furthermore, classical risk factors such as smoking, alcohol consumption, and HPV infection may also contribute to cancer development in patients under the age of 45 years. Local recurrences were the most common site of recurrence, regardless of tumour location and treatment modality. Therefore, future study designs in this patient cohort should potentially investigate intensified treatment approaches.
